# Risk Factors Associated With Hospitalization, Severity, and Mortality From COVID-19 Among Children and Adolescents in Mexico

**DOI:** 10.7759/cureus.107407

**Published:** 2026-04-20

**Authors:** Francisco Vargas Hernandez, Alexandra E Soto Piña, José J Garduño-García, Fernando Bastida Gonzalez, Alejandra D Benitez Arciniega, Luis R Garcia Cortes, Diana L Perez Cabrera, Samantha W Martínez Angeles, Caroline C Pulido-Alvarado, Elizabeth Alderete Flores

**Affiliations:** 1 Family Medicine, Faculty of Medicine, Universidad Autonoma del Estado de Mexico, Toluca de Lerdo, MEX; 2 Diabetes and Endocrinology, Faculty of Medicine, Universidad Autonoma del Estado de Mexico, Toluca de Lerdo, MEX; 3 Diabetes and Endocrinology, Faculty of Medicine, Universidad Autónoma del Estado de México, Toluca de Lerdo, MEX; 4 Allergy and Immunology, Laboratorio de Biologia Molecular Estatal, Instituto de Salud del Estado de Mexico, Toluca de Lerdo, MEX; 5 Family Medicine, Coordinación Auxiliar Médica de Investigación en Salud, OOAD México Oriente, Instituto Mexicano del Seguro Social, Tlalnepantla de Baz, MEX; 6 General Practice, Unidad de Medicina Familiar No. 64 “Tequesquinahuac”, Instituto Mexicano del Seguro Social, Tlalnepantla de Baz, MEX; 7 Pediatrics, Faculty of Medicine, Universidad Autonoma del Estado de Mexico, Toluca de Lerdo, MEX

**Keywords:** children and adolescents, covid-19, covid-19 disease, covid-19 severity, hospitalization, outcomes in hospitalized covid-19 patients

## Abstract

Introduction

Mexico has seen a high number of COVID-19 cases and deaths among children. The majority of cases were in Mexico City and the State of Mexico. There is heterogeneity in the clinical and epidemiological parameters of risk or protection at the national and international levels in this age group. The objective of the study is to identify the epidemiological and clinical factors associated with severe COVID-19, hospitalization, pneumonia at admission, and mortality among children and adolescents.

Materials and methods

A retrospective, analytical, and multicenter study based on hospital patients was conducted between 2020 and 2023 in four general and regional hospitals of the Mexican Social Security Institute (IMSS), Mexico. A total of 1,698 subjects diagnosed with COVID-19, aged between one and 17 years of both sexes, were included. Multiple binary logistic regressions were performed and stratified by laboratory diagnosis, obtaining simple and adjusted ORs for the clinical and epidemiological variables associated with severe COVID-19.

Results

The model adjusted and stratified by laboratory diagnosis for COVID-19 severity showed that obesity had an OR of 2.46 (95% CI, 1.37-4.43), being under five years of age had an OR of 2.01 (95% CI, 1.43-2.84), headache had an OR of 2.00 (95% CI, 1.18-3.39) and dyspnea had an OR of 1.82 (95% CI, 1.06-3.11). In the unadjusted analysis, male sex was associated with an OR of 1.82 (95% CI, 1.4-2.2) for severity.

Conclusion

The results suggest that being under five years of age, being overweight or obese, and experiencing cough, headache, and dyspnea are risk factors for COVID-19 complications in the hospital setting, based on clinical diagnosis. These findings have very limited applicability to mild community-acquired cases. Prospective cohort studies are needed to confirm these findings and include pediatric anthropometric standards.

## Introduction

In 2020, the World Health Organization (WHO) declared COVID-19 a public health emergency [1]. Older adults and those with comorbidities have been identified as the groups most vulnerable to severe forms of the disease [2]. Cases among children and adolescents account for 1% to 5% of all reported cases, and the risk of developing severe or critical illness is lower compared to adults [3].

Globally, there have been 697 million cumulative cases and more than six million deaths from COVID-19, of which more than 32,000 were in children 0-14 years old by 2023 [4]. In Mexico, 502,726 cases have been confirmed in children under 18 years of age as of 2023, and the highest prevalence of COVID-19 in the country was reported in Mexico City and the State of Mexico [5]. According to the Mexican Department of Health, a high number of COVID-19 cases has been reported in children and adolescents under 19 years of age in the State of Mexico since 2023 [6,7]. In 2021 and 2022, COVID-19 mortality in children and adolescents increased to 4.4% and 3.7% for children aged 0-9 and 10-19, respectively [8].

Mexican children under two years of age showed higher rates of hospitalization, intensive care admission, and mortality compared to older children during six pandemic waves between 2020 and 2023 [9]. Furthermore, cardiovascular disease, hypertension, diabetes, and immunosuppression were identified as major risk factors for severe outcomes in the pediatric population. These findings emphasize the importance of age-stratified risk assessment and tailored public health strategies for children in Mexico [9].

Most children and adolescents present mild or asymptomatic forms of COVID-19; however, some can develop serious outcomes such as pediatric multisystem inflammatory syndrome (MIS-C), acute respiratory distress syndrome (ARDS), and death [10,11]. The epidemiological conditions associated with severe COVID-19 in children and adolescents have been studied since the beginning of the pandemic [12]. Although most pediatric cases present mild COVID-19, up to 19% may present moderate disease, 6% critical disease, and a mortality rate of 0.03% to 3.3% [13]. Some risk factors for severe disease in this age group are age, sex, obesity, overweight, diabetes, immunocompromised states, and the presence of comorbidities [14].

There are some clinical parameters whose protective or risk performance has not been fully established, or is controversial in Mexican children and adolescents. For example, variables like age, overweight or obesity, signs and symptoms of COVID-19 have not been associated with disease severity, hospitalization, or death in children [15,16]. Likewise, the clinical usefulness of these parameters has not been fully associated with the development of severity, pneumonia upon admission, and mortality in children and adolescents with COVID-19.

Therefore, the primary objective of this study was to identify the epidemiological and clinical factors associated with severe COVID-19, hospitalization, pneumonia at admission, and mortality among children and adolescents aged 1 to 17 years with confirmed or clinically diagnosed COVID-19 at four hospitals in the eastern region of the State of Mexico. Specifically, we evaluated the independent associations of age (<5 years), sex, and overweight/obesity with these outcomes using logistic regression analysis.

## Materials and methods

Study design and participants

This was a retrospective, analytical, and multicenter study based on hospital patients. Information was obtained from secondary sources (sociodemographic, epidemiological, and clinical data) on children and adolescents with COVID-19 who were treated in the Eastern region of the State of Mexico at four general and regional hospitals of the Mexican Social Security Institute (IMSS).

Children and adolescents of both sexes who required hospitalization and/or outpatient follow-up, 1-17 years old, with a confirmed diagnosis of COVID-19, with normal weight, grade I and II obesity [17] were included. Subjects with malnutrition or underweight [18], psychomotor delay, oncological diseases, end-stage renal disease, and cystic fibrosis were excluded. Incomplete data in physical or digital files was eliminated.

Data collection

**Figure 1:**
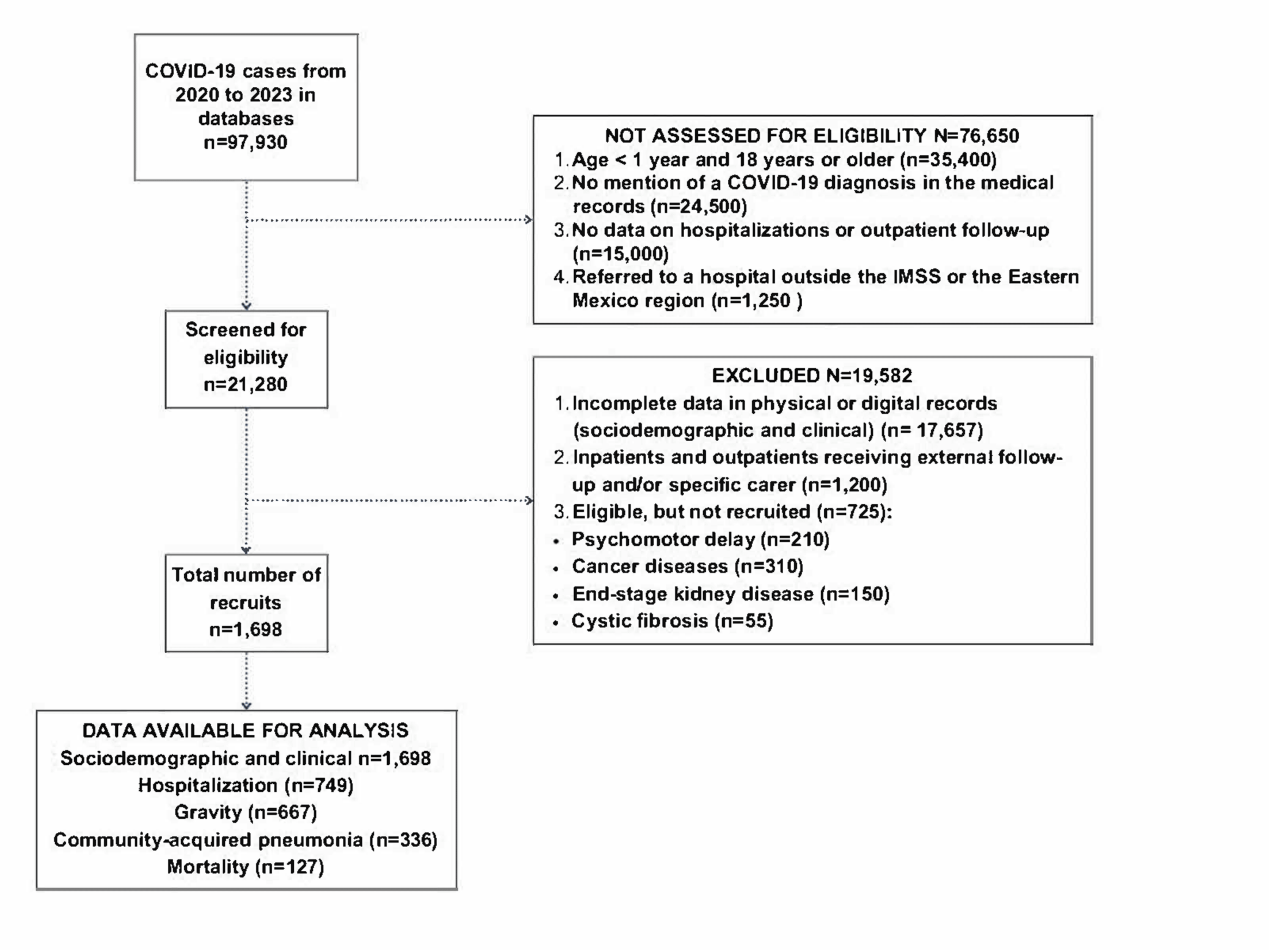
STROBE flow diagram Flowchart of COVID-19 patients in the Eastern Region of the State of Mexico. STROBE: Strengthening the Reporting of Observational Studies in Epidemiology; IMSS: Mexican Social Security Institute Image credit: The figure was created by the authors using Canva (Canva Pty Ltd., Sydney, Australia; version 2026).

From an initial database of 97,930 individuals of all ages diagnosed with COVID-19 between 2020 and 2023, 21,280 patients remained after excluding records outside the age range, those without a documented diagnosis, and those without clinical follow-up. Subsequently, exclusion criteria were applied, resulting in a total of 1,698 participants included in the analysis. Sociodemographic and clinical variables, as well as hospitalization, outpatient follow-up, severity, pneumonia, and mortality, were assessed in these participants (Figure 1).

The database of the Online Notification System for Epidemiological Surveillance (SINOLAVE) and physical records from four general and regional hospitals in the eastern IMSS zone in the State of Mexico were reviewed from April 2020 to December 2023. Children and adolescents who had been vaccinated against COVID-19 between 2021 and 2023 were excluded. Weight and height were extracted from the official records of the hospitals. There were 1,698 files of subjects who met the inclusion criteria for analysis. All participants were analyzed as a series of cases.

Sociodemographic, clinical, and epidemiological data, as well as data on clinical outcomes (severity, hospitalization, nasal oxygen therapy on admission, intubation, and mortality), were collected by the principal investigator and three associate investigators, who visited the Medical Information and Clinical Records departments (ARIMAC) of four regional and local general hospitals belonging to the IMSS’s Office of Coordination and Administration for Eastern Mexico (OOAD). The researchers who collected the data had previously received training on the standardization of clinical conditions (exposure factors) and clinical outcomes (clinical results) to ensure their correct classification.

Study definitions: severe COVID-19 and overweight or obese

Severe COVID-19 was defined according to the classification of the WHO and the U.S. Centers for Disease Control and Prevention (CDC), as severe illness (respiratory rate >90%, SpO_2_ <94%, pulmonary infiltrates >30%) and critical illness (respiratory failure, septic shock, and multiple organ dysfunction). Non-severe cases were classified as mild disease (signs or symptoms of COVID-19, without respiratory distress or abnormal chest imaging) and moderate disease (clinical or radiological lower respiratory tract disease, but with SpO_2_ >94%).

Overweight and obesity in the pediatric population were defined based on standardized anthropometric criteria adjusted for age and sex. According to the WHO, overweight is defined as a Z-score > +1 (equivalent to the 85th percentile) and obesity as a Z-score > +2 (equivalent to the 97th percentile) [19].

Statistical analysis

The statistical analysis was performed using IBM SPSS Statistics for Windows, Version 27 (Released 2019; IBM Corp., Armonk, New York, United States). For qualitative variables (being under five years of age, sex, symptoms, severity, hospitalization, pneumonia upon admission, and mortality), frequencies and percentages were determined. For the quantitative variable (age), the type of distribution was determined using statistical testing criteria, using the Kolmogorov-Smirnov test (p>0.05), asymmetry (-0.5, -0.5), and kurtosis (-0.2, -0.2). The median and interquartile ranges (IQR 25, 75) were used for the age variable. Age was categorized as a dichotomous variable (≥ or <5 years) for the purpose of conducting risk analyses. Epidemiological and clinical factors associated with hospitalization, COVID-19 severity, pneumonia at admission, and mortality were analyzed using simple binary logistic regression to obtain odds ratios (ORs) with 95% confidence intervals (CIs) and p-values. A p-value <0.05 was considered statistically significant.

A multiple binary logistic regression model was performed for COVID-19 severity. Variables were considered to have biological plausibility, statistically significant associations, or a tendency toward increased risk of severity. The following were included: male sex, being under five years of age, overweight or obesity, presence of cough, dyspnea, headache, and fever were considered as covariates. The following were obtained: ORs with 95% CI, p-values, Nagelkerke's R^2^, and the Hosmer-Lemeshow test. In addition, a multiple binary logistic regression model was constructed, stratified by reverse transcription polymerase chain reaction (RT-PCR) and rapid antigen test results, to assess the severity of COVID-19, taking into account the following covariates: overweight or obesity, age under five years, cough, headache, fever, dyspnea, and male sex, yielding ORs, 95% CIs, and p-values. This is to obtain more accurate risk assessments based on the diagnosis.

Simple imputation was performed for missing data related to the subjects' sociodemographic, clinical, and epidemiological characteristics, with a missing rate of less than 20%. No imputation was performed for the diagnosis of COVID-19 or for clinical outcomes (severity, hospitalization, intubation, need for assisted ventilation (NAC), and mortality).

Ethical considerations

This project was approved by the Local Health Research Committee 1408 and the Research Ethics Committee, obtaining registration number R-2024-1408-029.

## Results

Out of the 1,698 subjects, 61.7% were female. The median age was seven years (IQR 3, 10). A total of 76% were children under five years of age, and the prevalence of obesity and overweight was 67.1%. The level of education was elementary school for 40.2%. Some subjects (52.2%) received the seasonal trivalent influenza vaccine. Fever, cough, and headache were present in 65.5%, 52.9%, and 52.2%, respectively. Moreover, 18.3% and 24.1% of the subjects tested positive for COVID -19 by RT-PCR nasopharyngeal and rapid antigen test, respectively. Pneumonia was observed on admission in 19.8% of cases, 39.3% were severe cases of COVID-19, 44.1% of subjects were hospitalized, and 6.7% required endotracheal intubation. The mortality rate was 7.5% (Table 1).

**Table 1:** Sociodemographic and clinical characteristics of children and adolescents with COVID-19 in the eastern region of the State of Mexico n: total number of study subjects; IQR: interquartile ranges; BMI: body mass index; RT-PCR: reverse transcription polymerase chain reaction

Parameter	n = 1698, n (%)
Age, median, IQR (25,75)	7 (3, 10)
Age under five years	1293 (76)
Gender	
Female	1048 (61.7)
Education level	
Not in school	205 (12.2)
Preschool in progress	340 (20.0)
Elementary school in progress	683 (40.2)
Secondary in progress	250 (14.7)
High school in progress	200 (11.8)
Overweight or obesity	1138 (67.0)
Trivalent influenza vaccine administered	886 (52.2)
Signs and symptoms of COVID-19	
General	
Fever	1112 (65.5)
General malaise	815 (48.0)
Irritability	391 (23)
Headache	887 (52.2)
Respiratory	
Odynophagia	558 (32.9)
Cough	899 (52.9)
Rhinorrhea	520 (30.6)
Chills	3 (28.4)
Nasal congestion	71 (4.2)
Hoarseness	12 (0.7)
Dyspnea	291 (17.1)
Cyanosis	72 (4.2)
Anosmia	60 (3.5)
Coryza	40 (2.4)
Dysgeusia	52 (3.1)
Osteoarticular	
Myalgia	679 (40.0)
Arthralgia	635 (37.4)
Prostration	81 (4.8)
Low back pain	1 (0.1)
Chest pain	203 (12)
Abdominal pain	270 (15.9)
Diarrhea	198 (11.7)
Type of COVID-19 diagnostic test	
RT-PCR nasopharyngeal swab	311 (18.3)
Rapid antigen	410 (24.1)
Operational definition	977 (57.5)
Contact with COVID-19	
Family member	1241 (74.4)
External	436 (26.6)
Place of contact with a person with COVID-19	
Home	1241 (74.4)
Outside the home	436 (26.6)
Pneumonia upon hospital admission due to COVID-19	336 (19.8)
Endotracheal intubation due to COVID-19	113 (6.7)
Severity of COVID-19	667 (39.3)
Hospitalization for COVID-19	749 (44.1)
COVID-19 mortality	127 (7.5)

Regarding COVID-19 severity, an OR of 1.8 (95% CI (1.4-2.2)) and p = 0.044 were obtained for males. Age under five years had an OR of 1.3 (95% CI (1.1-1.7)) and p = 0.034. Overweight or obesity showed an OR of 7.6 (95% CI (5.8-10)) and p = 0.001. Furthermore, the variables associated with hospitalization were found to be male sex, which had an OR of 1.8 (95% CI (1.4-2.1)) and p = 0.05, and age under five years, which had an OR of 5.9 (95% CI (4.8-7.4)) and p = 0.05 and the presence of overweight or obesity had an OR of 1.31 (95% CI 1.1-1.6)) and p = 0.030. In addition, the parameters associated with pneumonia on admission were male sex with an OR of 7.2 (95% CI (5.7-9.3)) and p = 0.05, age under five years with an OR of 6.3 (95% CI (5.3-7.5)) and p = 0.006, and overweight or obesity with an OR of 3.8 (95% CI (2.8-5.3)) and p = 0.001. Finally the parameters associated with mortality were male sex with an OR of 4.1 (95% CI (3.3-5.1)) and p = 0.05, age under five years with an OR of 1.7 (95% CI (1.1-2.8)) and p = 0.026, as well as overweight or obesity with an OR of 3.4 (95% CI (2.1-5.7)) and p = 0.001 (Table 2).

**Table 2:** Factors associated with COVID-19 complications in children and adolescents in the eastern region of the State of Mexico ^a ^Simple logistic binary regression; OR: odds ratio, CI: confidence interval

Parameter	COVID-19 severity (n = 667)		Hospitalization (n = 749)		Pneumonia on admission (n = 336)		Mortality (n = 127)	
	OR with 95% CI	p-value^a^	OR with 95% CI	p-value^a^	OR with 95% CI	p-value^a^	OR with 95% CI	p-value^a^
Male (Ref.: female)	1.8 (1.4-2.2)	0.044	1.8 (1.4-2.1)	0.05	7.2 (5.7-9.3)	0.05	4.1 (3.3-5.1)	0.05
Being under five years of age (Ref.: age 6 or older)	1.3 (1.1-1.7)	0.034	5.9 (4.8-7.4)	0.05	6.3 (5.3-7.5)	0.006	1.7 (1.1-2.8)	0.026
Overweight or obese (Ref.: not overweight or obese)	7.6 (5.8-10)	0.001	1.3 (1.1-1.6)	0.030	3.8(2.8-5.3)	0.001	3.4 (2.1-5.7)	0.001
Presence of cough (Ref.: absence of cough)	1.3 (0.5-1.9)	0.80	1.2 (0.5-1.5)	0.79	1.5 (0.5-1.6)	0.09	2.2 (1.5-3.4)	0.001
Fever (Ref.: absence of fever)	1.1 (0.6-1.5)	0.384	1.1 (0.8-1.2)	0.942	1.0 (0.5-1.3)	0.001	1.2 (0.7-1.5)	0.765

The significant variables in the model adjusted for severe COVID-19 were obesity or being overweight, being under five years of age, and the presence of dyspnea, cough, and headache (Table 3).

**Table 3:** Adjusted model: sociodemographic and clinical factors associated with severe COVID-19 in children and adolescents OR: odds ratio; CI: confidence interval; r^2^: coefficient of determination; Ref.: reference category

Logistic regression	Factor	OR (95% CI)	p-value
r^2^ of Nagelkerke = 0.25; Hosmer-Lemeshow test = 10.23; p = 0.24	Overweight or obese (Ref: not overweight or obese)	8.06 (6.04-10.75)	<0.01
	Dyspnea (Ref.: absence of dyspnea)	2.36 (1.70-3.28)	0.01
	Being under five years of age (Ref.: age 6 or older)	2.01 (1.43-2.84)	0.01
	Presence of cough (Ref.: absence of cough)	1.64 (1.23-2.12)	0.02
	Headache (Ref.: absence of headache)	1.45 (1.08-1.95)	0.02
	Male (Ref.: female)	1.15 (0.82-1.51)	0.3
	Fever (Ref.: absence of fever)	1.10 (0.81-1.50)	0.5

The model adjusted and stratified according to RT-PCR and rapid antigen test results for COVID-19 severity showed that being overweight or obese, being under five years of age, headache, and dyspnea are risk factors (Table 4).

**Table 4:** Adjusted model stratified by laboratory test results (RT-PCR and rapid antigen test): sociodemographic and clinical factors associated with severe COVID-19 in children and adolescents OR: odds ratio; CI: confidence interval; r^2^: coefficient of determination; Ref.: reference category; RT-PCR: reverse transcription polymerase chain reaction

Logistic regression	Factor	OR (95% CI)	p-value
r^2^ of Nagelkerke = 0.15; Hosmer-Lemeshow test = 9.83, p = 0.27	Overweight or obese (Ref: not overweight or obese)	2.46 (1.37-4.43)	<0.01
	Being under five years of age (Ref.: age 6 or older)	2.01 (1.43-2.84)	0.01
	Dyspnea (Ref.: absence of dyspnea)	1.82 (1.06-3.11)	0.02
	Headache (Ref.: absence of headache)	2.00 (1.18-3.39)	0.03
	Male (Ref.: female)	1.46 (0.94-2.28)	0.09
	Presence of cough (Ref.: absence of cough)	1.01 (0.61-1.67)	0.96
	Fever (Ref.: absence of fever)	1.15 (0.71-1.86)	0.55

## Discussion

In this study, our data revealed that factors such as being overweight or obese, male gender, and being under five years of age are potential risk factors for COVID-19 complications among participants, primarily in a hospital setting and diagnosed based on clinical or epidemiological criteria.

In this study, men were more likely to develop severe COVID-19. This finding may be explained by lower production of type I alpha interferon (IFN-I) and reduced inhibition of SARS-CoV-2 replication [20,21]. This makes male sex one of the most important factors for complications of COVID-19 [21,22]. Similarly, a study in adults over 18 years of age showed that male sex is a risk factor for unfavorable outcomes in COVID-19. This is consistent with other findings, such as a higher frequency of hospitalization, need for intensive care, and mortality in adult and elderly men [21,22].

The research findings show that children under five years of age had a lower risk of hospitalization, pneumonia, severe illness, and mortality. It is important to note that the innate immune system undergoes functional reprogramming up to age 21. This mechanism may be involved in protection against COVID-19 complications. School attendance and frequent respiratory infections may drive functional reprogramming of innate immune system cells, preparing the host against future respiratory infections, such as SARS-CoV-2 [23]. The development of the innate and adaptive immune responses can play a role in this respect, including incomplete vaccination schedules, and lower frequencies of recurrent respiratory and gastrointestinal infections that improve the innate immune system [24].

This is consistent with other clinical studies that report a lower frequency of hospitalization and a lower risk of developing severe COVID-19 and complications [25,26]. However, it is different from other reports in children where the percentages of severe and critical cases were 10.6%, 7.3%, 4.2%, 4.1%, and 3.0% for the age groups <1, 1 to 5, 6 to 10, 11 to 15, and >15 years, respectively.

A third important risk factor for severe COVID-19 and hospitalization was the presence of overweight or obesity associated with severe COVID-19. The risk of developing SARS-CoV-2 disease is higher in people with obesity (46%) than in people with normal weight [27]. In the presence of obesity, central adiposity can affect respiratory muscles by inflammation and decrease in lung capacity, leading to an increase in angiotensin-converting enzyme 2 (ACE2) expression and chronic inflammation [21]. In addition, obesity is a risk factor for intubation and severe or critical illness in pediatric patients with COVID-19 [28]. In a case-control study with children from 1 to 18 years of age, obesity was the most important risk factor for susceptibility and severity of COVID-19, above male sex and age, with a higher number of cases with complications [29].

Furthermore, overweight and obesity are associated with chronic adipopathy involving systemic inflammation, endothelial dysfunction, and decreased nitric oxide [30]. These conditions can worsen the prognosis of children and adolescents with severe COVID-19. Other research revealed an increase in plasma glucose levels following SARS-CoV-2 infection [31], suggesting a metabolic alteration possibly linked to a synergic inflammatory state induced by both obesity and SARS-CoV-2. These findings are also consistent with the onset of hyperglycemia in adult subjects with COVID-19 without a history of diabetes [32]. This may involve a risk of developing diabetes related to stress hyperglycemia and preexisting adipopathy [33].

In Mexico, there is a high prevalence of overweight and obesity among children. This, combined with unhealthy lifestyles, poverty, and lack of access to healthcare services and vaccines, can be considered risk factors for severe COVID-19 in this population. Therefore, clinical follow-up programs should be implemented during both the acute phase and the post-COVID-19 phase, with monitoring of body composition, blood glucose, and liver biomarkers [34].

Future studies on the interaction between clinical and genetic factors are essential to explain the complications observed in children with COVID-19. The high prevalence of obesity among Mexican children may be determined in part by genetic and epigenetic conditions [35,36]. These conditions partly explain the susceptibility to multifactorial diseases and the variability in clinical outcomes found in this study [37,38].

Some social determinants and access to health care contribute to morbidity and mortality from COVID-19 in children and adolescents in the State of Mexico. The disorderly and dispersed population growth in the State of Mexico limits access to health services for all age groups. Urban and rural areas with higher poverty rates and less access to health services, such as in the State of Mexico, contribute to higher infant mortality rates of 34.4% and under-five mortality rates of 3.6% in Mexico [39]. Social determinants of health were not directly measured, limiting the ability to assess their contribution to the observed associations.

Certain social determinants and access to healthcare contribute to the burden of COVID-19 in children and adolescents in the State of Mexico. Disorderly and dispersed population growth in the State of Mexico limits access to health services for all age groups [36]. Social determinants of health, which were not directly measured in this study, limit the ability to assess their impact on the associations found.

Limitations

One of the limitations of this study was that COVID-19 diagnosis via RT-PCR and rapid antigen tests was performed in less than half of the participants, which may result in a higher number of suspected cases without actual diagnostic confirmation. Similarly, the use of a combined classification for overweight and obesity results in a high prevalence of the condition in the study. This is due to the lack of detailed anthropometric parameters in the medical records, which prevented distinguishing between overweight and obesity; therefore, their combined frequency was reported. This explains the 67.1% prevalence and the overall impact of excess weight. It is acknowledged that the high mortality rate found in this study may be influenced by a Berkson bias.

It is acknowledged that the study design (retrospective and conducted in four hospitals) does not allow for the establishment of a causal relationship, but only associations.

Vaccination data were not systematically recorded in medical records, so it was not possible to include them in this study; however, more than half of the participants were children under five years of age with limited access to vaccines in Mexico, and authorization from the Mexican Ministry of Health only started in 2022. The exclusion of underweight and malnourished children may have introduced a susceptibility bias, which could have overestimated the association between overweight/obesity and the severity of COVID-19.

Strengths

This study included a large multicenter sample of 1,698 participants from four IMSS hospitals in eastern Mexico State. This constitutes a significant strength in the context of a middle-income country and provides reasonable statistical power for the logistic regression analysis. Furthermore, from a public health perspective, practical risk factors (young age <5 years, male sex, overweight/obesity, headache, cough, and dyspnea) were examined in a population with high rates of childhood obesity. The findings are consistent with the general literature on pediatric COVID-19 in Latin America and reflect the actual conditions of the pandemic in Mexican public hospitals. Furthermore, the use of simple and multiple binary logistic regression to estimate adjusted ORs is methodologically sound for identifying independent associations. A multivariate analysis stratified by laboratory tests was also conducted for the outcome of severe COVID-19, which provides greater certainty in our reported results.

## Conclusions

The results suggest that being under five years of age, being overweight or obese, and experiencing cough, headache, and dyspnea are risk factors for COVID-19 complications in the hospital setting, based on clinical diagnosis. These findings have very limited applicability to mild, community-acquired cases. Prospective cohort studies are needed to confirm these findings and include pediatric anthropometric standards. It is proposed that these parameters be integrated into the prognostic assessment of this age group. It is also recommended to intensify well-child follow-up strategies and promote lifestyle changes among children and adolescents in primary care centers in Mexico.
